# Phenotypic Changes of Peripheral γδ T Cell and Its Subsets in Patients With Coronary Artery Disease

**DOI:** 10.3389/fimmu.2022.900334

**Published:** 2022-07-08

**Authors:** Yan Li, Silin Jiang, Jiawei Li, Mengzhuo Yin, Fuxin Yan, Yuyuan Chen, Yan Chen, Tongwei Wu, Mengliang Cheng, Yihua He, Hongbin Liang, Hang Yu, Qingqing Qiao, Zhigang Guo, Yan Xu, Yanan Zhang, Zheng Xiang, Zhinan Yin

**Affiliations:** ^1^ National Center for International Research of Bio-targeting Theranostics, Guangxi Key Laboratory of Bio-targeting Theranostics, Collaborative Innovation Center for Targeting Tumor Diagnosis and Therapy, Guangxi Talent Highland of Bio-targeting Theranostics, Guangxi Medical University, Nanning, China; ^2^ Guangdong Provincial Key Laboratory of Tumor Interventional Diagnosis and Treatment, Zhuhai Institute of Translational Medicine Zhuhai People’s Hospital Affiliated with Jinan University, Jinan University, Zhuhai, China; ^3^ The Biomedical Translational Research Institute, Faculty of Medical Science, Jinan University, Guangzhou, China; ^4^ Department of Geriatrics, Guangzhou First People’s Hospital, School of Medicine, South China University of Technology, Guangzhou, China; ^5^ Department of Cardiology, Nanfang Hospital, Southern Medical University, Guangzhou, China; ^6^ Department of Medicine Ultrasonics, Nanfang Hospital, Southern Medical University, Guangzhou, China; ^7^ Department of Neurology, Nanfang Hospital, Southern Medical University, Guangzhou, China; ^8^ Department of Cardiology, Huiqiao Medical Center, Nanfang Hospital, Southern Medical University, Guangzhou, China

**Keywords:** coronary atherosclerotic heart disease, atherosclerosis, NKG2D, γδ T cells, Vδ2^+^T cell, Vδ2^-^T cells

## Abstract

Coronary atherosclerotic heart disease (CAD) is a chronic inflammatory cardiovascular disease with high morbidity and mortality. Growing data indicate that many immune cells are involved in the development of atherosclerosis. However, the immunological roles of γδ T cells in the initiation and progression of CAD are not fully understood. Here, we used flow cytometry to determine phenotypical changes of γδ T cells and their subpopulations in peripheral blood samples collected from 37 CAD patients. The Pearson correlation coefficient was used to analyze the relationship between the clinical parameter (serum LDL-C level) and the changes of immunophenotypes of γδ T cells. Our results demonstrated that the frequencies and absolute numbers of total γδ T cells and Vδ2^+^ T cells were significantly decreased in CAD patients when compared to healthy individuals. However, the proportion of Vδ1^+^ T cells was much lower in CAD patients than that of healthy individuals. Most importantly, a significant alteration of the Vδ1/Vδ2 ratio was found in CAD patients. In addition, a series of surface markers that are associated with costimulatory signals (CD28, CD40L, CD80, CD86), activation levels (CD69, CD25, HLA-DR), activating NK cell receptors (NKp30, NKp46, NKG2D) and inhibitory receptors (PD-1, CTLA-4, PD-1, Tim-3) were determined and then analyzed in the total γδ T cells, Vδ2^+^T cells and Vδ2^-^T cells of CAD patients and healthy individuals. The data demonstrated that immunological activities of total γδ T cells, Vδ2^+^T cells, and Vδ2^-^T cells of CAD patients were much lower than those in healthy individuals. Moreover, we found that there were positive correlations between the serum LDL-C levels and frequencies of CD3^+^γδ^+^ T cells, CD69^+^Vδ2^+^T cells, NKG2D^+^Vδ2^+^T cells, and NKp46^+^Vδ2^+^T cells. By contrast, there was an inverse correlation between the levels of serum LDL-C and the frequencies of CD69^+^Vδ2^-^T cells and NKp46^+^Vδ2^-^T cells. Accordingly, these findings could help us to better understand the roles of γδ T cells in the CAD, and shed light on the development of novel diagnostic techniques and therapeutic strategies by targeting γδ T cells for CAD patients.

## Introduction

Cardiovascular diseases (CVDs) continue to increase in prevalence and deaths worldwide, and remain the leading cause of severe disease burden and death in the world (1). Coronary atherosclerotic heart disease (CAD) is one of the most common cardiovascular diseases as well as the one of the leading causes of death in middle-aged and elderly people (2). The main pathophysiological change of CAD is coronary artery atherosclerosis (AS), which is a chronic inflammatory disease involved with many large and medium-sized arteries. Severe coronary stenosis and/or unstable atherosclerotic plaque rupture can lead to vascular embolism, myocardial ischemia, angina pectoris, myocardial infarction, arrhythmia, and even sudden death, which seriously threatens human life and health. The occurrence and development of atherosclerotic heart disease are related to a variety of risk factors, and metabolic factors, such as hypertension and hyperlipidemia (1). In order to reduce the occurrence of various complications, premature death and disability caused by coronary artery disease, more studies are required to investigate the underlying mechanisms of occurrence and development of CAD and coronary atherosclerosis and this will help us to explore new strategies for prevention of atherosclerosis and CAD.

The formation of atherosclerosis is complex and multifactorial, in which immunological dysregulation and serious inflammatory response are suggested to be the critical keys to its occurrence and progression. The various immune cells, such as monocytes, macrophages, dendritic cells, NK cells of the innate immune system as well as T cells and B cells of the adaptive immune system, are supposed to be involved in all processes of formation and development of atherosclerosis and rupture of vulnerable plaques. Some immune cells that infiltrate into the atherosclerotic plaque and the interactions of cells induce the secretion of pro-inflammatory cytokines which maintain the atherosclerotic plaque in an inflammatory microenvironment (3). The immunological roles of monocytes, macrophages and CD4^+^T cells, which are the most common T cells in plaques and atherosclerotic lesions, have been extensively studied. However, the immunological functions of some other immune cells in atherosclerosis are still unclear, such as γδ T cells (4). γδ T cells are innate-like T cells that carry TCR γ and δ chains. Unlike αβT cells, γδ T cells only account for 1-10% of T cells in human peripheral blood but are abundant in mucosal tissues such as skin, intestine, and lung (5). γδ T can recognize, in a major histocompatibility complex (MHC) non-restricted (6) manner, endogenous and exogenous phosphorylated antigens (pAgs) such as (E)-4-hydroxy-3-methyl-but-2-enyl pyrophosphate (HMBPP) and isopentenyl pyrophosphate (IPP) (7) and respond rapidly as the first line of immune defense. γδ T cells can also be activated through their surface-expressed TCRs and natural killer cell receptors, such as NKp30, NKp44, and natural-killer group 2 member D (NKG2D) (6). Activated γδ T cells can exhibit multiple immunological functions by expressing and producing cytotoxins such as TRAIL, Fas/Fas-L, granzyme B and perforin. In addition, γδ T cells can also produce large amounts of cytokines, such as IFN-γ, TNF-α, and IL-17. Human γδ T cells can generally be divided into two major subpopulations based on the expression of the TCR Vδ chain in the peripheral blood: Vδ2^+^T cells and Vδ2^-^T cells (8). Accordingly, 65~90% of γδ T cells in adult peripheral blood are Vδ2^+^T cells, which are almost always paired with Vγ9^+^, thus these cells are often referred to as Vγ9^+^Vδ2^+^T cells (9). Vγ9^+^Vδ2^+^T cells usually display a cytotoxic phenotype and secrete excessive amounts of IFN-γ and TNF-α, which have the potent killing effects against many hematological tumors and solid tissue tumors (10). While Vδ2^-^T cells are mainly Vδ1^+^T cells, with fewer Vδ3^+^T and Vδ5^+^T cells (9). Unlike Vγ9^+^Vδ2^+^T cells, this Vδ2^-^T cell subpopulation mainly exists in mucosal tissues, and only a small part (less than 30%) exists in peripheral blood. In addition, Vδ1^+^ T cells mainly play an immunosuppressive role. Previous studies reported that Vδ1^+^ T cells can induce FoxP3 expression in the presence of TGF-β and IL-2/IL-15, and their immunomodulatory effects are similar to those of Treg cells (11, 12). A large body of evidence indicates that γδ T cells are regarded as a bridge between innate and adaptive immune responses, which play an important role in the human immune response (13).

The presence of γδ T cells in human atherosclerotic plaques was first reported in 1993 (14). However, there are still only a few studies to mention the immunological functions of γδ T cells in atherosclerosis. The evidence which was collected from an experimental model of atherosclerosis demonstrated that lack of γδ T cells decreased plasma total cholesterol levels and reduced atherosclerosis in the aortic sinus of ApoE^-/-^ TCRδ^-/-^ mice, although these differences did not reach statistical significance (15). Additionally, a significant increase in γδ T cells, which produce IL-17 but not IFN-γ, was also found in the aortic root and arch of ApoE KO mice, and depletion of these cells reduced the size of early atherosclerotic lesions at this site in mice (16). However, another TCRδ^-/-^ApoE^-/-^ mouse study found no critical role for γδ T cells in the development of early atherosclerosis in the total aorta of mice after 10 weeks of high-fat diet feeding (17). These studies suggest a pathogenic role for γδ T cells in early atherosclerosis in mice and their effects may be site-specific. Recent studies demonstrated that IL-23R^+^γδ T cells are frequently found in the aortic root of Ldlr^-/-^Il23rgfp/^+^ mice. Moreover, the absence of this subset of γδ T cells reduces reduced the formation of AS lesions in the aortic root. And the γδ T cells were confirmed by scRNAseq to be the predominant cells expressing IL-23R and IL-17A in the aorta (18). This suggested that the pathogenic role of γδ T cells in early mouse AS may be related to IL-17A- and IL-23R-mediated immune response. Although some progress has been made with γδ T cells in experimental mouse models, the specific pathogenic mechanisms of γδ T cells in early atherosclerotic lesions and how they change with disease progression have not been fully elucidated, and it is even more challenging to translate these findings in animal models to human disease. At present, the roles of γδ T cells in the development of human atherosclerotic disease are less studied and remain largely unknown. A Multi-Ethnic Study of Atherosclerosis (MESA) cohort study showed that a higher proportion of γδ T cells in older adults was associated with poorer cardiac function in a subclinical state with cardiovascular risk factors but without heart failure (19). Analysis of high-throughput gene expression datasets revealed that the proportion of infiltrating γδ T cells in human AS plaques was decreased and significantly negatively correlated with inflammation-related pathways such as the IL-23 signaling pathway and NOTCH2 signaling pathway (3). This study found that γδ T cells from patients with acute myocardial infarction (AMI) had restricted expression of γδ rearrangement of TCR and higher expression of IL-17A, suggesting that γδ T cells may play an important role in the pathological progression of AMI (20). These results suggested that γδ T cell-mediated inflammatory responses may play an important role in the formation and development of human coronary atherosclerotic heart disease; however, the immunological functions of γδ T cells in advanced atherosclerosis has not been elucidated.

Therefore, based on the pathogenic role of γδ T cells in early atherosclerosis in mice, our study intends to analyze and compare the changes in the absolute number and immunophenotypes of γδ T cells and their subpopulations in the peripheral blood of CAD patients and healthy individuals, as well as the analysis of the correlation between clinically relevant indices and the immunophenotypes in the peripheral blood of CAD patients. In this study, we demonstrated that immunological characteristics of total γδ T cells, Vδ2^+^T cells, and Vδ2^-^T cells exhibited significant alteration in CAD patients when compared with the healthy individuals. Most interestingly, our data found that serum LDL-C level had a diametrically opposite correlation with the frequencies of subpopulation cells in Vδ2^+^T cells and Vδ2^-^T cells, particularly the cells that expressed CD69. These results could provide us with more clues and hints to reveal the potential functions of γδ T cells in the progression of CAD.

## Materials and Methods

### Patients and Samples

Including 24 male cases and 13 female cases, 37 CAD patients with 37~82 years old were enrolled who have been diagnosed with coronary angiography in the Department of Cardiology, Nanfang Hospital of Southern Medical University from April to June 2021. To assess the phenotypes of peripheral immune cells, 10 mL of heparinized anticoagulant was collected before coronary stenting in CAD patients. The clinical and demographic characteristics of CAD patients were described in **Table 1**. At the same time, 84 healthy individuals aged 40 to 66 years were recruited for this study, including 47 males and 37 females. The exclusion criteria of the healthy individuals included (1) a history of cardiovascular system diseases such as myocardial infarction, heart failure, angina pectoris, and/or cerebrovascular disease (2); hypertension, diabetes, and obesity; (3) severe liver damage, severe infectious diseases, hematological or autoimmune diseases. Similarly, 10 mL of peripheral blood was collected for subsequent immunological assays. Ethical approval was obtained from the Medical Ethics Committee of Nanfang Hospital of Southern Medical University.

**Table 1:** Clinical and demographic characteristics of CAD patients.

	CAD (n=37)
Age (years)	64.70 ± 9.1
Sex (male/female)	24/13
Smoking	15 (40.5%)
Hypertension	20 (54.1%)
Diabetes	8 (21.6%)
Gensini	39.88 ± 36.89
LDL-C (mmol/L)	2.79 ± 0.93
HDL-C (mmol/L)	1.14 ± 0.28
TG (mmol/L)	1.66 ± 0.83

LDL-C, Low-density lipoprotein cholesterol; HDL-C, High-density lipoprotein cholesterol; TG, Triglyceride.

### Peripheral Immune Cell Phenotype Analyzing by Flow Cytometry

To determine the phenotypic surface markers of γδ T cells and their subpopulations, collected peripheral blood samples were analyzed by flow cytometry. After the collected heparinized anticoagulant whole blood (1 mL) was directly lysed twice with red blood cell lysis solution, the whole blood leukocytes were isolated, and then stained with mouse anti-human fluorescein-conjugated monoclonal antibodies against different markers (**Supplementary Table 1**). After incubation in the dark for 15 mins, the cells were washed twice with PBS. Subsequently, the cells were resuspended in 200 uL PBS, and then added 5 uL of CountBright™ Absolute Counting Beads (Thermo Fisher Scientific). Finally, samples were collected using the instrument FACSVerse (BD biosciences) and data were analyzed using FlowJo 10.5.3 (FlowJo LLC).

### Statistical Analysis

Data were processed and analyzed using Microsoft Excel 2019 and Graphpad Prism 8.0.1. Unpaired t-test was used for statistical comparison of immune indexes between CAD patients and healthy individuals, and the Pearson correlation coefficient was used to calculate the correlation between two continuous variables of clinical related indexes of CAD patients and immune indexes in their peripheral blood. Statistical graphs were drawn by Graphpad Prism 8.0.1, all tests were set as two-tailed, P<0.05 was considered to be significantly different, and the results in the figure were expressed as Mean with SD.

## Results

### Changes in the Proportion of γδ T Cells and Their Subsets in Peripheral Blood of CAD Patients

**Figure 1:**
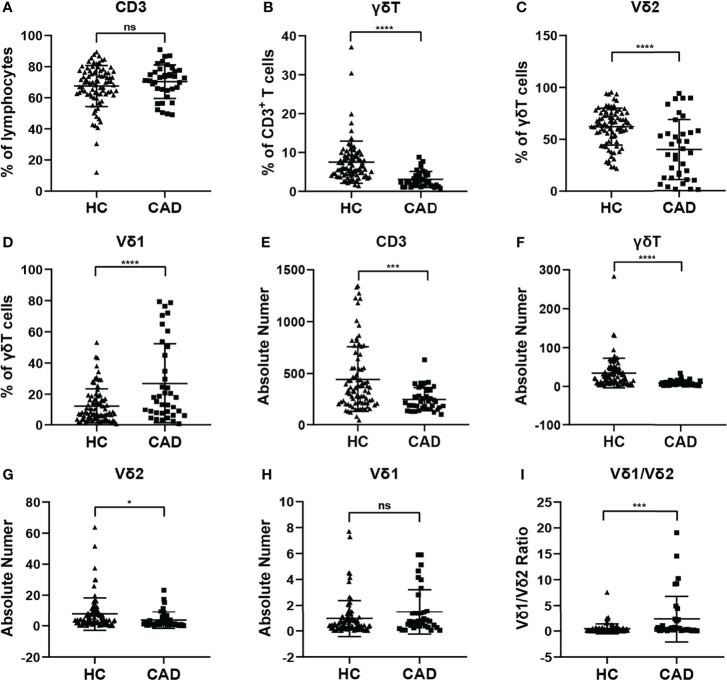
Statistical comparison of the percentages and absolute numbers of γδ T cell and its subsets Vδ1+T cells, Vδ2+T cells, and Vδ1/Vδ2 ratios in the peripheral blood between healthy individuals (HC) and CAD patients. **(A)** CD3^+^T cells, **(B)** γδ T cells, **(C)** Vδ2^+^T and **(D)** Vδ1^+^T cells frequencies as percentages of lymphocytes, CD3^+^T cells, and total γδ T cells respectively, absolute numbers of **(E)** CD3^+^T cells, **(F)** γδ T cells, **(G)** Vδ2^+^T and **(H)** Vδ1^+^T cells and **(I)** Vδ1/Vδ2 ratios of healthy individuals versus CAD patients. *P < 0.05, ***P < 0.001, and ****P < 0.0001; ns, no significance.

To investigate whether the immunological features of γδ T cells and their subsets were altered in CAD patients. The percentages and absolute numbers of γδ T cells and their subsets in the peripheral blood of CAD patients and healthy individuals were analyzed, respectively. The gating strategy identifying γδ T cells, Vδ1^+^ and Vδ2^+^ γδ T cell subsets were shown in **Supplementary Figure 1A**, and the results indicated that the percentages of CD3^+^T cells (**Figure 1A**), γδ T cells in CD3^+^ T cells (**Figure 1B**), Vδ2^+^T cells in γδ T cells (**Figure 1C**) in the CAD patients were significantly decreased when compared to that in healthy individuals. However, the CAD patients had a higher frequency of Vδ1^+^T cells in γδ T cells than that in healthy individuals (**Figure 1D**). When further analyzing the absolute number of CD3^+^T cells (**Figure 1E**), γδ T cells in CD3^+^ T cells (**Figure 1F**), the data indicated that Vδ2^+^T cells in γδ T cells (**Figure 1G**) were higher in CAD patients than those in healthy individuals. By contrast, there was no significant difference in the absolute number of Vδ1^+^T cells in γδ T cells between CAD patients and healthy individuals (**Figure 1H**). Moreover, the Vδ1/Vδ2 ratio in CAD patients was elevated (2.326 ± 4.448), when compared to that in healthy individuals (0.411 ± 0.944) (**Figure 1I**).

### The Activation Levels of γδ T Cells in the CAD Patients Are Altered

**Figure 2:**
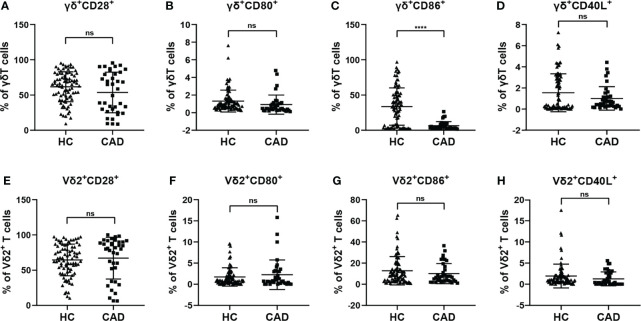
Expression of co-stimulatory molecules on the surface of γδ T cell and its subsets in peripheral blood of healthy individuals (HC) and CAD patients **(A, E)** CD28, **(B, F)** CD80, **(C, G)** CD86, and **(D, H)** CD40L frequencies as percentages of total γδ T cells, and Vδ2^+^T cells respectively, of healthy individuals versus CAD patients. ****P < 0.0001; ns, no significance.

γδ T cells can exhibit multiple immunological functions because this population of cells can express CD28, CD80/CD86, and CD40L. In addition, CD80/CD86-CD28 and CD40-CD40L signaling are involved in regulating different functions of γδ T cells and their subsets. To investigate the functional status of γδ T cells during CAD, we next compared the frequencies of CD28, CD80, CD86, and CD40L positive cells in total γδ T cells, and Vδ2^+^T cells between CAD patients and healthy individuals. The gating strategy for the identification of γδ T cells, Vδ2^+^T cells, and Vδ2^-^T cells, and their surface immunophenotypes were presented in **Supplementary Figure 1B**. Analysis of γδ^+^ CD28^+^ T cells (**Figure 2A**), γδ^+^ CD80^+^ T cells (**Figure 2B**), and γδ^+^ CD40L^+^T cells (**Figure 2D**) frequencies demonstrated that there were no significant differences between CAD patients and healthy individuals. However, the frequency of γδ^+^ CD86^+^ T cells in total γδ T cells is much higher in healthy individuals compared to CAD patients (**Figure 2C**). In the Vδ2^+^T cells, the percentage of CD28^+^ T cells, CD80^+^ T cells, CD86^+^ T cells, and CD40L^+^ T cells were found no significant differences between healthy individuals and CAD patients (**Figures 2E–H**).

**Figure 3:**
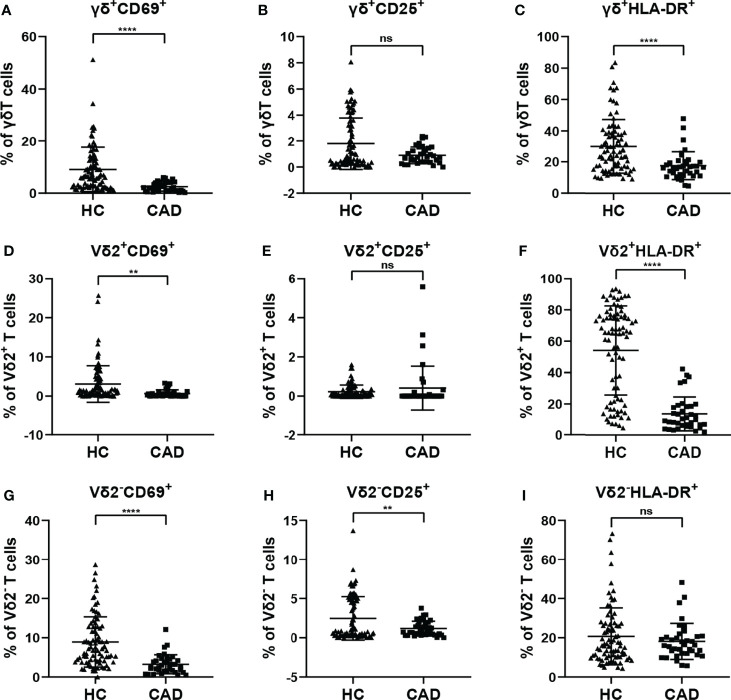
Expression of activation marker on the surface of γδ T cell and its subsets in peripheral blood of healthy individuals (HC) and CAD patients. **(A, D, G)** CD69, **(B, E, H)** CD25, and **(C, F, I)** HLA-DR frequencies as percentages of total γδ T cells, Vδ2^+^T, and Vδ2^-^T cells respectively, of healthy individuals versus CAD patients. **P < 0.01 and ****P < 0.0001; ns, no significance.

To investigate the changes in the activation status of γδ T cells and their subsets in CAD, the activation markers of γδ T cells and their subsets were also determined for evaluating their potential immunological roles. Activated γδ T cells can express several classical markers, including CD69, CD25, and HLA-DR. Additionally, in human peripheral γδ T cells, the majority subset of γδ T cells is Vδ2^+^T cells, whereas γδ T cells expressing other Vδ elements (Vδ2^-^T cells) are rare in the blood but they still display some functions. Therefore, the CD69^+^, CD25^+^ and HLA-DR^+^ cells in total γδ T cells, Vδ2^+^T cells, and Vδ2^-^T cells were compared between healthy individuals and CAD patients. Our results indicated that the proportions of total γδ T cells, Vδ2^+^T cells, and Vδ2^-^T cells that were CD69^+^ were significantly lower in CAD patients compared to healthy individuals (**Figures 3A, D, G**). In addition, no significant differences were found in the percentages of CD25^+^ cells in the total γδ T cells and Vδ2^+^T cells between healthy individuals and CAD patients (**Figures 3B, E**). But in the Vδ2^-^T cells, the frequency of CD25^+^ cells were lower in CAD patients than that in healthy individuals (**Figure 3H**). Furthermore, the frequencies of HLA-DR^+^ cells in total γδ T cells and Vδ2^+^T cells were lower in CAD patients compared to healthy individuals (**Figures 3C, F**). And there was no significant difference in the percentage of HLA-DR^+^ cells in Vδ2^-^T cells between healthy individuals and CAD patients (**Figure 3I**).

**Figure 4:**
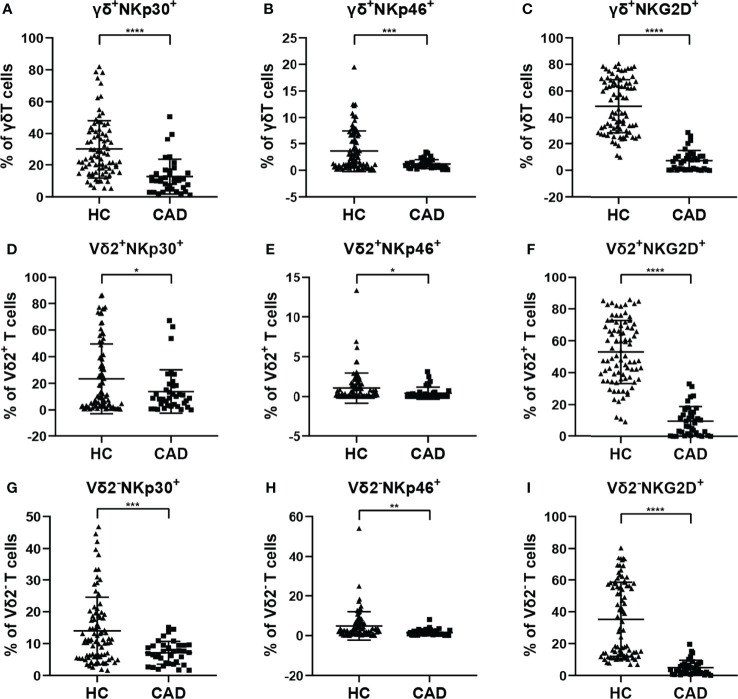
Expression of NK cell-activating receptors on the surface of γδ T cell and its subsets in peripheral blood of healthy individuals (HC) and CAD patients. **(A, D, G)** NKp30, **(B, E, H)** NKp46, and **(C, F, I)** NKG2D frequencies as percentages of total γδ T cells, Vδ2^+^T, and Vδ2^-^T cells respectively, of healthy individuals versus CAD patients. *P < 0.05, **P < 0.01, ***P < 0.001, and ****P < 0.0001.

According to previous studies, the activating NK cell receptors, such as NKp30, NKp46, and NKG2D, play critical roles in modulating multiple functions of γδ T cells. To determine the phenotypic changes of activating NK cell receptors in γδ T cells and their subsets in CAD, the surface levels of NKp30, NKp46, and NKG2D in total γδ T cells, Vδ2^+^T cells, and Vδ2^-^T cells were compared between healthy individuals and CAD patients. The percentages of NKp30^+^ (**Figures 4A, D, G**), NKp46^+^(**Figures 4B, E, H**), and NKG2D^+^ cells (**Figures 4C, F, I**) in total γδ T cells, Vδ2^+^T cells, and Vδ2^-^T cells were significantly lower in CAD patients compared to healthy individuals.

### Changes in the Expression of Immunosuppressive Molecules on the Surface of γδ T Cells in Peripheral Blood of CAD Patients

**Figure 5:**
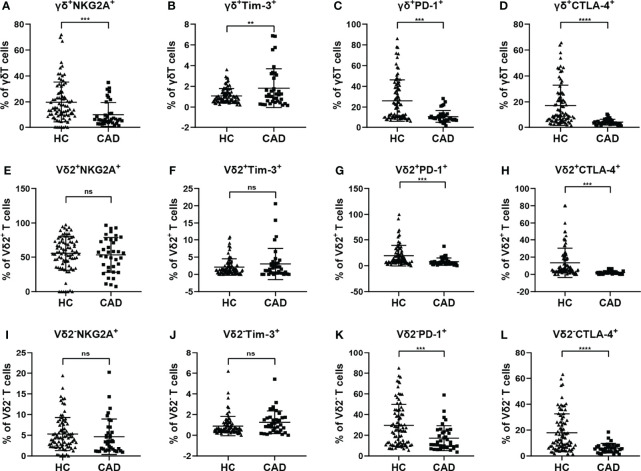
Expression of immunosuppressive molecules on the surface of γδ T cell and its subsets in peripheral blood of healthy individuals (HC) and CAD patients. **(A, E, I)** NKG2A, **(B, F, J)** Tim-3, **(C, G, K)** PD-1, and **(D, H, L)** CTLA-4 frequencies as percentages of total γδ T cells, Vδ2^+^T, and Vδ2^-^T cells respectively, of healthy individuals versus CAD patients. **P < 0.01, ***P < 0.001, and ****P < 0.0001; ns, no significance.

Although CAD was previously known as a lipid accumulation-mediated disease, it has now been considered a chronic inflammatory disease. The multiple inhibitory receptors (including PD-1, CTLA-4, PD-1, and Tim-3) are involved in regulating the functions of γδ T cells in the inflammatory response. To better understand the roles of inhibitory signals in γδ T cells during the onset of CAD, the proportions of NKG2A^+^, Tim-3^+^, PD-1^+^, and CTLA-4^+^cells among total γδ T cells, Vδ2^+^T cells, and Vδ2^-^T cells were determined. Our results indicated that the frequencies of NKG2A^+^, CTLA-4^+^, and PD-1^+^ cells in the total γδ T cells were significantly lower in CAD patients (**Figures 5A, C, D**), but the frequencies of Tim-3^+^ cells in the total γδ T cells were significantly higher in CAD patients compared to healthy individuals (**Figure 5B**). In the Vδ2^+^T cells, the frequencies of PD-1^+^, and CTLA-4^+^ cells are much lower in CAD patients than that in healthy individuals (**Figures 5G, H**). And no significant differences in the percentages of NKG2A^+^, Tim-3^+^ cells between healthy individuals and CAD patients (**Figures 5E, F**). By contrast, there were no significant differences in the percentages of NKG2A^+^ and Tim-3^+^ cells in Vδ2^-^T cells between healthy individuals and CAD patients (**Figures 5I, J**). However, the frequencies of PD-1^+^ Vδ2^-^T cells (**Figure 5K**) and CTLA-4^+^ Vδ2^-^T cells (**Figure 5L**) were significantly lower in CAD patients than that in healthy individuals

### Correlation of Serum LDL-C With the Immunophenotype of γδ T Cells in CAD Patients

**Figure 6:**
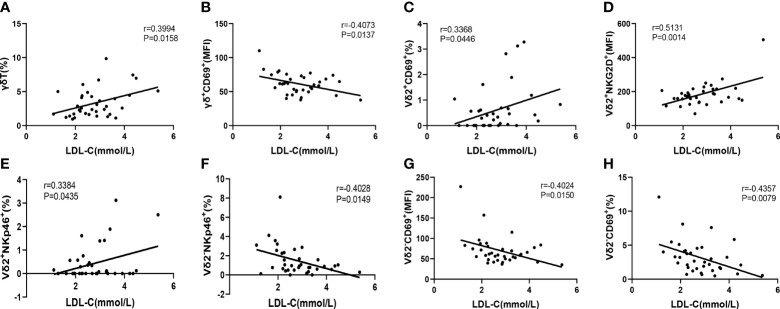
The Pearson correlation between serum LDL-C level in CAD patients and immune cell phenotypes of γδ T cell and its subsets in peripheral blood of CAD patients. **(A)** γδ T cells, **(B)** γδ^+^CD69^+^T cells, **(C)** Vδ2^+^CD69^+^T cells, **(D)** Vδ2^+^NKG2D^+^T cells, **(E)** Vδ2^+^NKp46^+^T cells, **(F)** Vδ2^-^NKp46^+^T cells and **(G, H)** Vδ2^-^CD69^+^T cells.

It is well known that elevated low-density lipoprotein cholesterol (LDL-C) level is a major risk factor for CAD. Therefore, we next to further evaluate the correlation of immunological features of γδ T cells and their subsets with the levels of serum LDL-C, which will help us to use these immunological factors as indicators to predict the development of CAD. The results indicated that the level of serum LDL-C in CAD patients was significantly positively correlated with the percentage of γδ T cells in total CD3^+^ cells (r=0.3994, P= 0.0158) (**Figure 6A**), However, the level of serum LDL-C was significantly negatively correlated with the expression of CD69 (r=-0.4073, P=0.0137) on the surface of γδ T cells in peripheral blood (**Figure 6B**). In addition, the level of serum LDL-C in CAD patients was significantly positively correlated with the proportions of CD69^+^ cells (r=0.3368, P=0.0446), NKG2D^+^ cells (r=0.5131, P=0.0014), and NKp46^+^ cells (r=0.3384, P=0.0435); r=0.3368) in Vδ2^+^T cells (**Figures 6C–E**). By contrast, there was an inverse correlation between the serum levels of LDL-C and the frequencies of CD69^+^Vδ2^-^T cells (r=-0.4357, P=0.0079) and NKp46^+^Vδ2^-^T cells (r=-0.4028, P=0.0149) (**Figures 6F–H**). Taken together, our data suggested that activation levels of Vδ2^+^T cells and Vδ2^-^T cells might be associated with the development of CAD and these two populations of cells may exhibit different potential functions in CAD.

## Discussion

The pieces of evidence collected from the animal models suggest that γδ T cells display a critical pathogenic role in the early stage of atherosclerosis. However, whether human γδ T cells display similar roles in CAD remains unclear. Here, this study aims to preliminarily explore the changes in immunophenotypes of γδ T cells in human coronary atherosclerotic heart disease. In this study, we included 37 patients with a clinical diagnosis of CAD and severe stenosis with greater than 80% arterial vascular involvement as the study group and recruited 84 healthy individuals as controls. Flow cytometry was used to analyze the alterations in the absolute numbers and immunophenotypic changes of γδ T cells and their subsets in peripheral blood between the two groups. Furthermore, we also analyzed the correlation between clinically relevant indicators and the immune cell phenotype in CAD patients. We found that the absolute number of circulating γδ T cells in the peripheral blood of CAD patients was significantly lower than that of healthy individuals, which may be due to the significantly lower percentage of naïve γδ T cells and the marked significantly increase of Fas, which is a molecule that mediates apoptosis on the surface of γδ T cells in the peripheral blood of CAD patients (**Supplementary Figure 2**). The proportion of γδ T cell subsets, such as the Vδ2^+^T cells and Vδ1^+^T cells, in the peripheral blood of CAD patients, is unbalanced, which leads to an alteration in the ratio of Vδ1/Vδ2 in CAD patients. Compared with healthy individuals, the expression of activation markers on the surface of γδ T cells and their subsets in the peripheral blood of CAD patients were significantly lower than those of healthy individuals. In addition, the levels of surface immunosuppressive molecules PD-1 and CTLA-4 on the surface of γδ T cells and their subsets in the peripheral blood of CAD patients were also significantly decreased, but the expression of the inhibitory marker Tim-3 on the surface of γδ T cells was significantly increased. We also observed that the serum LDL-C level of CAD patients was significantly positively correlated with the percentage of γδ T cells and was also different significant correlations with the immunophenotypes of different subsets. These results suggest that under the continuous stimulation of the chronic inflammatory environment in CAD and the functions of γδ T cells may be progressively exhausted in a state of low activation and high inhibition, suggesting an important association of γδ T cells and their subpopulations with the pathophysiological processes of human CAD.

Roman Kleindienst et al. were the first time to report that γδ T cells existed in human atherosclerotic plaques, and the number of γδ T cells was the highest in the early atherosclerotic lesions when there were relatively few CD3^+^T cells, and its proportion was 9.7% in the transition zone between normal vascular intima and lipid streaks, and then gradually decreased to 6.6% and 4.3% in fat streak and atherosclerotic plaque (14). Using high-throughput analysis technology to analyze three gene expression databases related to CAD, a recent study demonstrated that the number of γδ T cells infiltrating human atherosclerotic plaques was lower than that in control groups. Meanwhile, the expression levels of immunological function-related genes including the chemokines (CCL5, CX3CL1, CXCL10) in CAD plaques were higher than those in the control group (3). Previous studies have shown that the phenotypic distribution of immune cells differs between carotid atherosclerotic plaques and blood, and the frequencies of CD4^-^CD8^-^T cells were more abundant in plaques than that in blood. Furthermore, T cells in the blood were mostly quiescent, whereas the coexistence of activated, pro-inflammatory and exhausted T cells in the same plaque suggested a progressive loss of T cell function during chronic and prolonged inflammatory responses (21). Thus, these findings will extend our understanding of the interconnectedness of migration, heterogeneity, and functional alterations of immune cells between atherosclerotic plaques and peripheral blood. Our results demonstrated that the proportion of γδ T cells in the peripheral blood of CAD patients was also significantly lower than that of healthy individuals. We also found that the absolute number of subset Vδ2^+^T cells in the peripheral blood of CAD patients was significantly decreased compared with it in the healthy individuals, and the number of Vδ1^+^T cells was significantly increased, resulting in an increased Vδ1/Vδ2 ratio. Generally, since Vδ2^+^T cells in peripheral blood are the predominant γδ T cells, the Vδ1/Vδ2 ratio in peripheral blood is less than 1. But, the phenotypes of γδ T cell subsets in CAD patients and healthy individuals are so different, indicating that the balance between Vδ1^+^T cells and Vδ2^+^T cells is very important for the maintenance of immune function in CAD patients. However, whether the different functions of these two subpopulations of γδ T cells lead to such opposite phenotypes in CAD patients remains to be further investigated.

The functional responses of T cells require the co-existence of antigen-stimulatory signals and co-stimulatory signals, and the integration and transmission of these signals require the participation of activating or inhibitory molecules expressed on the surface of T cells. The immunoglobulin-like CD28 family and the tumor necrosis factor receptor superfamily are the important co-stimulatory molecules on the surface of T cells. And the interaction of CD28 on the surface of T cells and CD80/CD86 on the surface of antigen-presenting cells (APCs) is the critical costimulatory signal for activating T cells. According to the previous studies, the data have shown that the CD28/CTLA-4-CD80/CD86 pathway plays an important role in accelerating the development of atherosclerotic lesions, and was considered as an important potential target in immune regulation of atherosclerosis. For example, the absence of CD80/86 costimulation significantly reduced the development of early hyper-cholesterol-induced atherosclerotic lesions in Ldlr^-/-^ mice. This data suggested that CD80 and CD86 molecules are involved in the regulation of the occurrence of atherosclerotic lesions and the initiation of antigen-specific T cell responses in atherosclerotic lesions (22, 23). In addition, the CD80/CD86 pathway is also a promising biomarker of atherosclerotic plaque vulnerability. Analysis of atherosclerotic plaques from human carotid endarterectomy revealed that the expression levels of costimulatory molecules CD80 and CD86 were closely related to plaque vulnerability (24). In CTLA-4-transgenic/Apoe^-/-^ mice, overexpression of CTLA-4 can significantly reduce the formation of atherosclerotic lesions and significantly inhibit the accumulation of macrophages and CD4^+^T cells in the plaque, and then regulate atherosclerosis by down-regulating the expression of costimulatory molecules CD80, CD86, and CD28 (25). PD-1 is another regulatory molecule induced and expressed upon T-cell activation that plays an important role in modulating immune responses and autoimmunity by binding to its ligand PD-L1/2. Some studies have shown that deficiency of PD-1/PD-L1 could accelerate the development of atherosclerosis (26, 27). Our results showed that the expression levels of CD86, PD-1, and CTLA-4 in the peripheral blood of CAD patients were significantly lower than those of healthy individuals, indicating that the co-stimulatory signal required for activation of γδ T cells in CAD patients may be down-regulated in the chronic inflammation environment which established in the CAD patients. These findings suggested that abnormalities in CD28/CTLA-4-CD80/CD86 and PD-1-mediated signaling pathways may be involved in the pathogenic mechanism of γδ T cells in the progression of CAD. Previous studies have shown that Tim-3 is highly expressed on the surface of circulating NK cells in patients with atherosclerosis, suggesting that Tim-3 is involved in the occurrence of atherosclerosis and may be associated with the microenvironmental inflammation of atherosclerosis. Furthermore, treatment of statins can reduce the expression proportion of Tim-3 on NK cells in patients with atherosclerosis (28, 29). The treatment of anti-Tim-3 monoclonal antibody in LDLr^-/-^ mice exhibited therapeutic effects in the acceleration of atherosclerotic plaque formation accompanied by an increase in the number of monocytes/macrophages and CD4^+^T cells and a decrease in the number of regulatory T cells and regulatory B cells (30). Tim-3 is also significantly increased in non-classical immune cells human artery vascular smooth muscle cells (HASMCs) and can inhibit platelet-derived growth factor-BB (PDGF-BB)-induced inflammatory response by inhibiting the activation of NF-kB, and limiting the expression levels of inflammatory cytokines IL-6 and TNF-α, suggesting that Tim-3 as a potential target for controlling atherosclerotic (31). In our study, our results indicated that Tim-3 on the surface of γδ T cells in the peripheral blood of CAD patients is significantly higher than that of healthy individuals, suggesting that the Tim-3^+^γδ T cells might be involved in the formation of atherosclerosis, and may be an alternative therapeutic target. Another study found that expression levels of Tim-3 in CD8^+^T cells in patients with atherosclerosis were significantly up-regulated than those in healthy individuals. Furthermore, the blockade of Tim-3 could lead to a down-regulation in the production of anti-atherosclerotic cytokines, while an increase in the production of pro-atherosclerotic cytokines TNF-α and IFN-γ, which could promote the development of atherosclerotic lesions (32). This evidence reported that expression patterns of CD86, PD-1, and Tim-3 were altered in the development of CAD, suggesting that these molecules exhibited critical roles in this disease. Accordingly, our results showed that similar patterns were found in the CD86, PD-1, and Tim-3 expressions in γδ T cells of CAD patients. These data will extend our understanding that not only NK cells and CD8^+^T cells are the effector immune cells but also the putative role of γδ T cells in the pathogenic mechanism in the CAD.

A recent study reported that there was a significant downregulation of CD69 mRNA level in peripheral blood leukocytes in the patient with the progression of atherosclerosis. Thus, CD69 expression was considered as an independent predictor of subclinical atherosclerosis (33). In our study, the expression levels of CD69 on the surface ofγδ T cells Vδ2^+^T cells, and Vδ2^-^T cells in the peripheral blood of CAD patients were significantly lower than those of healthy individuals. Moreover, the expression of HLA-DR, which is a marker to indicate a late stage of T cell activation, on the surface of γδ T cells and Vδ2^+^T cells in CAD patients was lower than that of healthy individuals. Additionally, the expression of CD25, which plays an important role in the formation of high-affinity IL-2 receptors and promotes the proliferation of T cells, on the surface of Vδ2^-^T cells is lower in CAD patients compared with healthy individuals. Finally, our data showed the expression levels of NKG2D, NKp30, and NKp46 on the surface of γδ T cells and their subsets in the peripheral blood of CAD patients were significantly lower than those of healthy individuals. Similar to our findings, a previous study demonstrated that LDL inhibited the activation and functions of human Vδ2^+^T cells, in terms of down-regulation of activation markers, such as NKG2D expression (34). Together, these results suggest that the activation levels of γδ T cells and their subpopulations in peripheral blood of CAD patients are significantly reduced in the severe CAD, which may impair the immune function of γδ T cells in atherosclerosis.

Serum LDL-C level is an important risk factor for CAD, and it can be oxidized and modified to ox-LDL *in vivo*. And this reactivity can promote a series of complex pathophysiological processes *in vivo* and plays an important role in the occurrence and development of atherosclerosis. Our results indicated that the level of serum LDL-C in CAD patients was significantly positively correlated with the percentage of γδ T cells in peripheral blood. A previous study demonstrated that cholesterol levels in γδ T cells were much higher than that in αβT cells. And highly activated cholesterol metabolism can regulate immunological activities of γδ T cells, indicating that γδ T cells can quickly reach the cholesterol checkpoint, which did a contribution to the hyper-activated phenotype of γδ T cells (35). Moreover, a previous study also found that human Vδ2^+^T cells expressed LDL receptor post activation and treatment of LDL-C resulted in inhibition of functions of Vδ2^+^T cells, which in turn down-regulated the CD69 expression and IFN-γ production (34). In contrast, we found that levels of serum LDL-C were significantly positively correlated with the percentage of γδ T cells in the peripheral blood of CAD patients. In addition, the level of serum LDL-C in CAD patients was also significantly positively correlated with the expression levels of NKp46, NKG2D, and CD69 on the surface of Vδ2^+^T cells. Therefore, these data indicate that there is still controversy about the link between the cholesterol metabolism and immune functions of γδ T cells, especially in the Vδ2^+^T cells. Meanwhile, our analysis demonstrated that there was significantly negatively correlated with the expression of CD69 on the surface of γδ T cells and Vδ2^-^T cells and the expression of NKp46 on the surface of Vδ2^-^T cells. Here, in line with our findings, one previous study showed that CD69 as an ox-LDL receptor in T cells and CD69 expression in circulating T cells correlate inversely with subclinical atherosclerosis in the patients (33). Accordingly, the evidence collected from CAD patients will help us to completely understand how functions of γδ T cell and its subpopulations are differently modulated by cholesterol metabolism. Most importantly, our *in vivo* data further implies a potential role by which cholesterol differentially regulates the pathogenic mechanisms of γδ T cell subsets in the progression of CAD.

However, the limitation of this study could not be neglected. First, further large sample size study is warranted to validate these findings. And the effects of age, sexual and other factors in impacting the immunological features of γδ T cell should be further evaluated in CAD. Second, in this study, we only determined the phenotypic changes of γδ T cell subsets in the peripheral blood of patients. The immunological features of γδ T cell and its subpopulations are rarely investigated in CAD and should be determined in future studies by using the samples collected from blood and atherosclerotic plaque of CAD patients. Third, we found the phenotypic changes of γδ T cell subsets by comparing the data collected from CAD patients and healthy individuals. In the future study, using samples from each CAD patient at different time points in disease progression will further help us to clarify the clinical significance of phenotypic alterations of γδ T cell subsets.

Taken together, our findings show that immunophenotypes of γδ T cell and its subsets are significantly changed in the CAD patients. In addition, the expression levels of some markers, especially the CD69, in Vδ2^+^T cells and Vδ2^-^T cells exhibit different association patterns with the serum LDL level. These data indicate that γδ T cell subsets play an important role in the progression of CAD. And Vδ2^+^T cells and Vδ2^-^T cells may display different functions involved in the development of CAD. Therefore, prospective research is needed to confirm the functional diversity of γδ T cell subsets in CAD. We propose that future studies should investigate the link between cholesterol metabolism and pathogenic roles of γδ T cell subsets in the heart diseases, thus evaluating the therapeutic potential and clinical significance of γδ T cells in the clinic.

## Data Availability Statement

The raw data supporting the conclusions of this article will be made available by the authors, without undue reservation.

## Ethics Statement

The studies involving human participants were reviewed and approved by Medicine Ethics Committee of Nanfang Hospital, Southern Medical University, Guangzhou, Guangdong Province, China. The patients/participants provided their written informed consent to participate in this study.

## Author Contributions

ZY, ZX, and YX designed the study and critically revised the manuscript; YL perform the experiments, data analysis, and manuscript writing; SJ, YYC, YC, YX, HY, and QQ contributed to the collection and processing of experimental samples and data analysis; FY and YZ contributed to the collection of patient samples and management of clinical information of patients. All authors contributed to the article and approved the submitted version.

## Funding

The present study was financially supported by the Guangzhou Planned Project of Science and Technology (202102080073), the National Natural Science Foundation of China (82070606), the Natural Science Foundation of Guangdong Province, China (2021A1515011441), the National Natural Science Foundation of China (32000616 and 31570898) and the Basic and Applied Basic Research Fund of Guangdong Province (2020A1515111203 and 2016A030313112). This work was also supported by the Scientific and Technological Innovation Major Base of Guangxi (NO. 2018-15-Z04), Guangxi Key Research and Development Project (No. AB20117001), Guangxi Science and Technology bases and talent special project (No. AD17129062).

## Conflict of Interest

The authors declare that the research was conducted in the absence of any commercial or financial relationships that could be construed as a potential conflict of interest.

## Publisher’s Note

All claims expressed in this article are solely those of the authors and do not necessarily represent those of their affiliated organizations, or those of the publisher, the editors and the reviewers. Any product that may be evaluated in this article, or claim that may be made by its manufacturer, is not guaranteed or endorsed by the publisher.

## Supplementary Material

Supplementary Figure 1 Overview of gating strategy used in flow cytometric analysis. **(A)** Gating strategy identifying γδ T cells, Vδ1^+^ and Vδ2^+^ γδ T cell subsets, **(B)** Gating strategy identifying γδ T cells, Vδ2^+^ T and Vδ2^-^ γδ T cell subsets, and their immunophenotypes.

Supplementary Figure 2 Statistical comparison of naïve γδ T cells and the expression of Fas on the surface of γδ T cells in peripheral blood of healthy individuals (HC) and CAD patients. The percentage of **(A)** γδ^+^CD45RO^-^CD62L^+^ T cells (naïve γδ T cells), and **(B)** γδ^+^Fas^+^ T cells of healthy individuals versus CAD patients. ^***^P < 0.001, and ^****^P < 0.0001.
