# Supplementary figures and images for: Phenotypic Changes of Peripheral γδ T Cell and Its Subsets in Patients With Coronary Artery Disease
Source: Front Immunol. 2022 Jul 8;13:900334. doi: 10.3389/fimmu.2022.900334 (PMC9304556; doi:10.3389/fimmu.2022.900334)

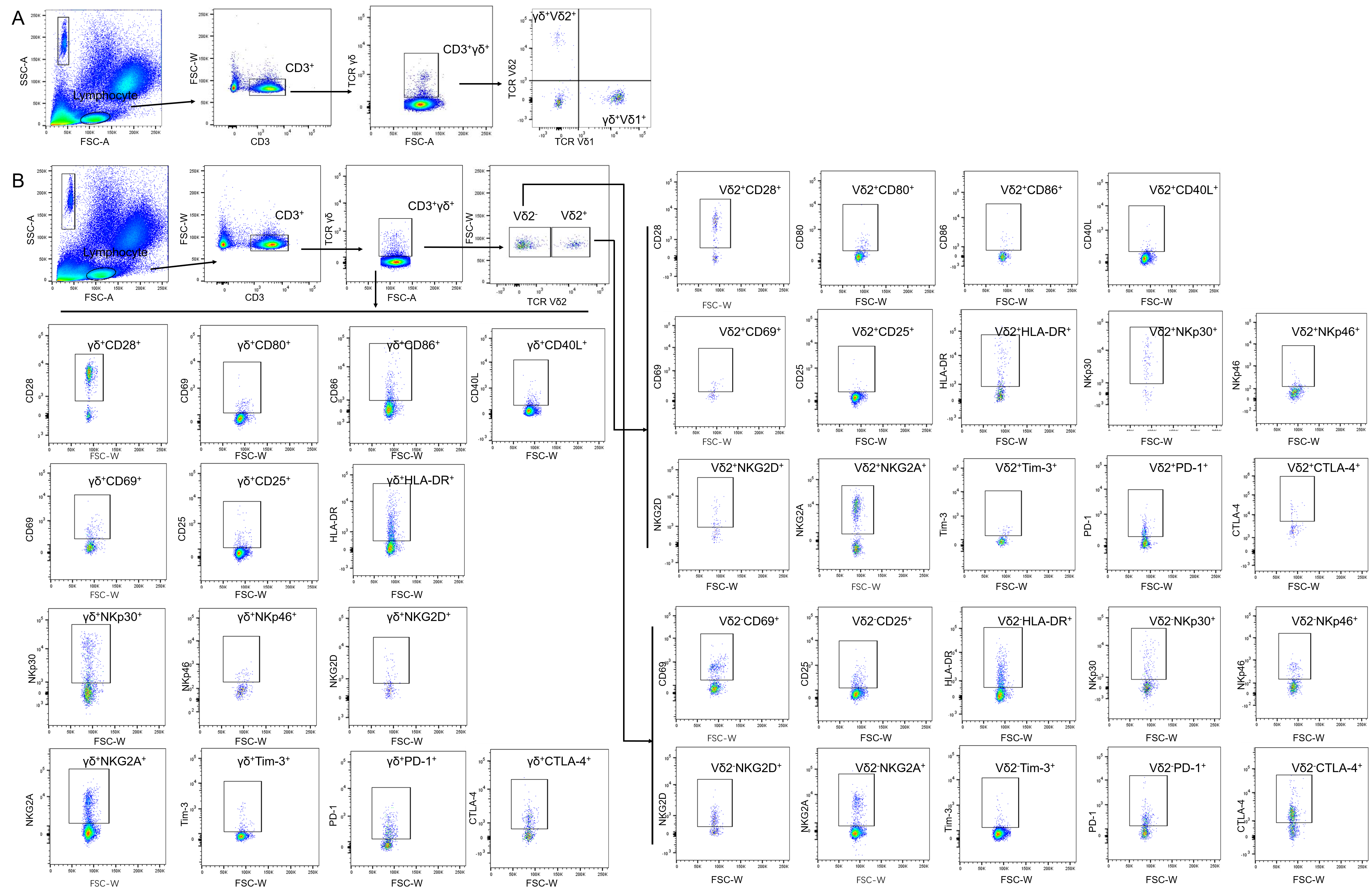

Supplement: Supplementary Figure 1 — Overview of gating strategy used in flow cytometric analysis. (A) Gating strategy identifying γδ T cells, Vδ1+ and Vδ2+ γδ T cell subsets, (B) Gating strategy identifying γδ T cells, Vδ2+ T and Vδ2- γδ T cell subsets, and their immunophenotypes. [file Image_1.tif]

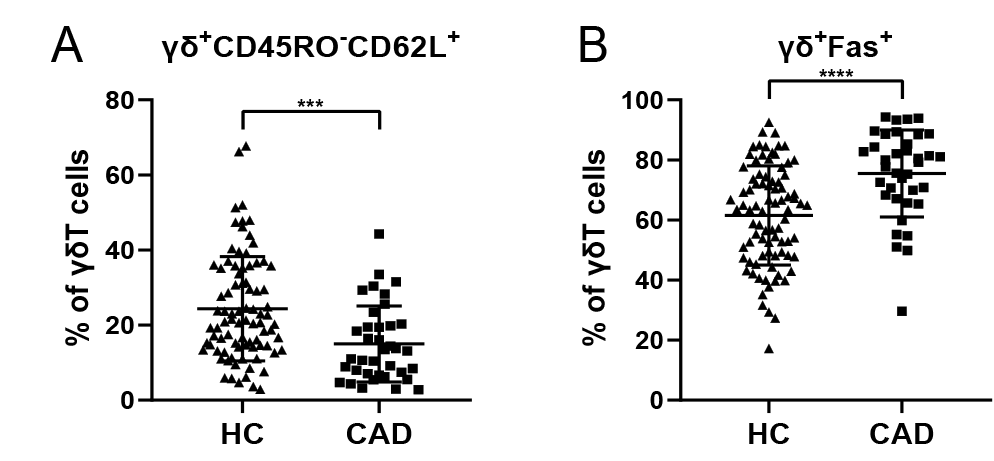

Supplement: Supplementary Figure 2 — Statistical comparison of naïve γδ T cells and the expression of Fas on the surface of γδ T cells in peripheral blood of healthy individuals (HC) and CAD patients. The percentage of (A) γδ+CD45RO-CD62L+ T cells (naïve γδ T cells), and (B) γδ+Fas+ T cells of healthy individuals versus CAD patients. ***P < 0.001, and ****P < 0.0001. [file Image_2.tif]
