# Supplementary material for: Phenotypic Changes of Peripheral γδ T Cell and Its Subsets in Patients With Coronary Artery Disease
Source: Front Immunol. 2022 Jul 8;13:900334. doi: 10.3389/fimmu.2022.900334 (PMC9304556; doi:10.3389/fimmu.2022.900334)
Supplement: Supplementary file 3 [file Table_1.docx]

**Supplementary Table**

**Supplementary Table 1. List of fluorescence conjugated antibodies with information about host species, clones, and companies.**

| Antibody | Fluorochrome | Host Species | Clone | Company |
| --- | --- | --- | --- | --- |
| CD3 | FITC | Mouse | OKT3 | Biolegend |
| CD3 | APC-Cy7 | Mouse | UCHT1 | Biolegend |
| CD3 | V500 | Mouse | UCHT1 | BD |
| TCR γ/δ | PE | Mouse | B1 | Biolegend |
| γδ TCR | BV421 | Mouse | 11F2 | BD |
| TCR Vδ2 | PE-Cy7 | Mouse | B6 | Biolegend |
| TCR Vδ2 | PerCP | Mouse | B6 | Biolegend |
| TCR Vδ1 | APC | Mouse | REA173 | Miltenyi Biotec |
| CD28 | PE-Cy5 | Mouse | CD28.2 | Biolegend |
| CD80 | FITC | Mouse | 2D10 | Biolegend |
| CD86 | APC | Mouse | IT2.2 | Biolegend |
| CD154(CD40L) | PE | Mouse | 24-31 | Biolegend |
| NKG2D | PerCP-Cy5.5 | Mouse | 1D11 | Biolegend |
| NKp30 | AF647 | Mouse | P30-15 | Biolegend |
| NKp46 | BV510 | Mouse | 9E2 | Biolegend |
| CD69 | FITC | Mouse | FN50 | Biolegend |
| CD25 | FITC | Mouse | BC96 | Biolegend |
| HLA-DR | PE-Cy5 | Mouse | L243 | Biolegend |
| NKG2A | APC | Mouse | S19004C | Biolegend |
| Tim-3 | BV510 | Mouse | F38-2E2 | Biolegend |
| PD-1 | BV421 | Mouse | NAT105 | Biolegend |
| CTLA-4 | PE-Cy7 | Mouse | L3D10 | Biolegend |
